# Association of Vegetable and Fruit Consumption with Urinary Oxidative Biomarkers in Teenaged Girls: A School-Based Pilot Study in Japan

**DOI:** 10.3390/ijerph191710474

**Published:** 2022-08-23

**Authors:** Yoshiko Sato, Ai Yamada, Masamitsu Miyanaga, Da-Hong Wang

**Affiliations:** 1 Department of Bioscience, Faculty of Life Science, Okayama University of Science, Okayama 700-0005, Japan; 2 Wakayama Shin–ai Junior and Senior High School, Wakayama 640-8151, Japan; 3 Japan Industrial Safety and Health Association, Osaka 550-0001, Japan

**Keywords:** vegetable/fruit consumption, oxidative biomarkers, hexanoyl-lysine, 8-hydroxy-2′deoxyguanosine, dityrosine, hydrogen peroxide, physical exercise, urine, teenaged girls

## Abstract

Hexanoyl-lysine (HEL), 8-hydroxy-2′deoxyguanosine (8-OHdG), and dityrosine (DT) have served as potential biomarkers for detecting oxidative modified lipids, DNA, and proteins in biological samples, respectively. Whether regular higher levels of consumption of vegetables/fruit (V/F) would decrease oxidative modification of these biomolecules in the body remain unelucidated. To examine the association of regular V/F consumption with the generation of these reactive oxygen species-induced biomarkers, this study evaluated V/F consumption in a school-based sample of teenaged girls (mean age 15.6 ± 1.7 years, *n* = 103), and quantified the formation of oxidative stress biomarkers in their urine. Only 19.4% and 23.3% of participants reported that they consumed the recommended daily amount of vegetables and fruits, respectively. Individuals who consumed lower levels of fruit (<100g/day) or vegetables (<250g/day) had significantly higher HEL excretion in their urine than those who consumed higher levels of fruit (≥100g/day) (*p* < 0.05) or vegetables (≥250g/day) (*p* = 0.057). The results of a multiple regression analysis showed that vegetable consumption was an important inhibiting factor of early lipid peroxidation measured as HEL in urine, independent of various confounders (β = − 0.332, *p* < 0.05). The findings suggest that relatively higher consumption of vegetables would help in the prevention of early lipid peroxidation in adolescents.

## 1. Introduction

Reactive oxygen species (ROS) are by-products of normal metabolic processes in all aerobic organisms. Under physiological conditions, the antioxidant defense systems in the body protect the cells against these species [1]. However, excess ROS generation can oxidatively modify the molecules of lipids, proteins, and DNA [1,2]. These modified molecules appear in biological samples as biomarkers and are indicators for early prediction of disease development and prevention [3,4,5]. For example, hexanoyl-lysine (HEL), 8-hydroxy-2′deoxyguanosine (8-OHdG), and dityrosine (DT) are known as ROS-induced products that have been considered as stable oxidative stress biomarkers for detecting lipid peroxidation/protein modification [6,7], DNA damage [1,8] and oxidation of protein, respectively. 

HEL is a stable oxidative stress biomarker for lipid peroxidation and protein modification [9]; Naito et al. reported increased HEL production by oxidatively modified low-density lipoprotein in the liver and kidneys of atherosclerotic rabbits [10]. In children with autism spectrum disorders, the urinary HEL adduct level was increased [11]. DT is one of the biomarkers of oxidation of protein [12]; urinary DT has been proposed to serve as a sensitive biomarker of the oxidation of protein. 8-OHdG is a biomarker of oxidative DNA damage [1,8]; elevated levels in urine have been observed in persons with diabetes mellitus [13], chronic renal failure [14], and cancer [15]. Exposure to environmental pollutants has been shown to increase 8-OHdG in body fluids, such as serum, urine, and bronchoalveolar lavage [16,17,18]. H_2_O_2_, a by-product of oxidative metabolism, is considered to be one of the ROS; it is able to diffuse across the cell membrane and form other highly reactive intermediates such as hydroxyl radical (OH) [19]. On the other hand, H_2_O_2_ is also considered as an inter- and intra-cellular signaling molecule acting as a “second messenger” to regulate the cellular processes in signal transduction cascades [20,21]. Urinary H_2_O_2_ levels are increased in patients with adult respiratory distress syndrome [22], Down’s syndrome [23], and diabetes mellitus [24].

The alterations in levels of the above-mentioned biomarkers have been studied in various pathological conditions, such as atherosclerotic lesions in animals [9,10], children with an autism spectrum disorder [11], diabetes mellitus [13], chronic renal failure [14], and cancer [15]. In healthy individuals, how the levels of biomarkers in the body are changed and whether they are associated with lifestyle factors have not yet been elucidated. In recent years, it has been suggested to use biomarkers to monitor the biological response to a lifestyle behavior, especially for V/F consumption [25].

Many research studies have indicated that the regular consumption of vegetables and fruit (V/F) is associated with a lower incidence of several common cancers, cardiovascular disease, and hypertension, by many potential mechanisms of action, including their protective role against ROS-induced oxidative lesions in the body [25,26,27]. In Japan, the average daily V/F consumption among teenagers (aged 15–19) remained low in 2017, at 272.2 g and 79.6 g, respectively, [28] compared with the recommended amounts of vegetables (350 g or more per day) and fruit (200 g or more per day) for better health [29,30]. Verhagen et al. reported that Brussels sprouts supplementation decreased the excretion of 8-OHdG in the urine of five non-smoking males [31]. Young et al. found a decreased level of the lipid peroxidation marker malondialdehyde (MDA) in the plasma of five volunteers during a 1500 mL fruit juice intervention [32]. Iwasawa et al. reported that urinary HEL and 8-OHdG levels in three volunteers was decreased on day two and on day four, respectively, by consuming Kiwi fruit three times a day for seven days [33]. However, all of these findings were based upon very small sample sizes. There are some studies that have found contradictory results: a randomized trial by Duthie et al. showed that a 12-week intervention with low V/F consuming adults showed a considerabe increase in their intake of fruits, fruit juices, and vegetables; however significant changes in antioxidant capacity and DNA damage were not observed [34]. Francina et al. reported no significant change in the urinary excretion of the lipid peroxidation marker (8-isoprostane) after a 12-week intervention of V/F intake [35]. In the literature, data on the association between V/F consumption and oxidative modification of biological molecules are unavailable in the adolescent population. To examine whether regular V/F consumption is associated with the generation of oxidative stress biomarkers in urine, the present study evaluated V/F consumption in a school-based sample of teenaged girls and quantified the formation of oxidative stress biomarkers in their urine. 

## 2. Subjects and Methods

### 2.1. Study Participants and Study Design

**Figure 1:**
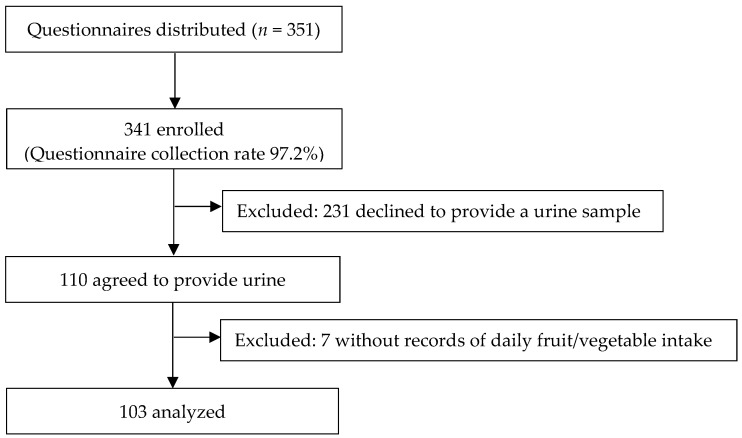
Flow chart of participant recruitment.

We recruited the participants from a local girls’ junior high and high school. A total of 351 students were invited to participate in the study and the study questionnaires were distributed to them. They were asked to answer questions about their daily fruit and vegetable intake, awareness of the current recommendations for V/F consumption, and their weekly physical exercise, among others. Three hundred and forty-one individuals answered the questionnaires (response rates: 97.2%), of which 231 were excluded because they declined to provide urine samples, and 7 were excluded due to missing responses to the daily fruit/vegetable intake questions. Finally, a total of 103 individuals (aged 12–18 years) who completed the questionnaires and agreed to provide urine samples were included (Figure 1). 

### 2.2. Estimation of Usual Vegetable and Fruit Intake

Data on daily V/F intake were obtained through a self-administered questionnaire, in which instructions and examples were presented in the questionnaire, for example, “One small bowl of vegetables is approx. 70g.”, “One medium apple or pear weighs approx. 200g” and “Two medium oranges or kiwis weigh approx. 200g”. Participants were asked to recall their daily average V/F intake by portion size and then to convert the portion size into grams of daily V/F intake (g/day), based on the calculating examples given in the questionnaire. The questionnaire also included information on demographic characteristics. The original questionnaire was kindly provided by Dr. Havas [36] and modified and tested for use in the Japanese population [37].

### 2.3. Urine Sampling and Determination of Oxidative Biomarkers in Urine

Morning spot urine (midstream urine) was collected from those who agreed to provide a urine sample, and was stored at −80 °C until analysis. The sample was dissolved and centrifuged at 2300× *g* (5000 rpm) for 5 min at 4 °C to remove any insoluble materials. 

Measurement of HEL in the urine was performed by a competitive enzyme-linked immunosorbent assay (ELISA), using a Hexanoyl-lysine analysis kit according to the manufacturer’s protocol (Japan Institute for the Control of Aging, Shizuoka, Japan) [7]. Using this method, the incubation of urine samples with the primary antibody (anti-HEl monoclonal antibody) was performed at 4 °C overnight. The antibodies bound to the HEL in the sample were washed 3 times with 0.05% Tween-20 in phosphate-buffered saline. Then, HRP-labeled anti-mouse IgG as a secondary antibody was added to the microtiter plate and incubated at room temperature (20–24 °C) for 1 h, and the unbound HRP-labeled secondary antibody was washed away. The amount of antibody bound to the plate was determined by the color developed from the addition of the chromogenic substrate 3,3′,5,5′-tetramethylbenzidine (TMB); the reaction was then terminated by adding 1M phosphoric acid and read at 450 nm with a microplate reader (SH-1200; Corona Electric Co., Ltd., Tokyo, Japan). All measurements were performed in duplicate on microtiter plates. 

Measurement of DT in the urine was carried out by a competitive ELISA using a Dityrosine ELISA kit according to the manufacturer’s protocol (Japan Institute for the Control of Aging, Shizuoka, Japan). In this method, the incubation of urine samples with the primary antibody (anti-DT monoclonal antibody) was performed at 4 °C overnight; the antibodies bound to the DT in the sample were washed 3 times with the washing buffer. Then, HRP-labeled anti-mouse IgG as a secondary antibody was added to the microtiter plate and incubated at room temperature (20–24 °C) for 1 h, and the unbound HRP-labeled secondary antibody was washed away. The amount of antibody bound to the plate was determined by the color developed from the addition of the chromogenic substrate TMB, and the reaction was then terminated and read at 450 nm [9] with the above-mentioned microplate reader. All measurements were performed in duplicate on microtiter plates.

Urinary 8-OHdG was determined by a competitive ELISA using a New 8-OHdG Check kit according to the manufacturer’s protocol (Japan Institute for the Control of Aging, Shizuoka, Japan). In this method, the incubation of the urine samples with the primary antibody (anti-8-OHdG monoclonal antibody) was performed at 37 °C for 1 h [38]; the antibodies bound to the 8-OHdG in the sample were washed 3 times with 0.05% Tween-20 in phosphate-buffered saline. An enzyme-labeled secondary antibody was then added to the microtiter plate and incubated at 37 °C for 1 h. After the unbound enzyme-labeled secondary antibody was washed away, the amount of antibody bound to the plate was determined by the color developed from the addition of a chromogenic substrate TMB, and the reaction was then terminated by adding 1M phosphoric acid. The absorbance was measured at 450 nm with the above-mentioned microplate reader. All measurements were performed in duplicate on microtiter plates.

The H_2_O_2_ in the urine was measured using the ferrous ion oxidation xylenol orange version*–*1 (FOX-1) assay with minor modification [24,39]. In brief, 20 µL of each urine sample was incubated with the same amount of catalase solution (2200 U/mL in 25 mM phosphate buffer, pH 7.0) or 25 mM phosphate buffer. Then, the samples were reacted with 160 µL of FOX-1 regent (100 µM xylenol orange, 100 mM sorbitol, 250 µM ammonium ferrous sulfate, and 25 mM H_2_PO_4_, pH adjusted to 1.7–1.8 by the addition of Na_2_HPO_4_) at 25 °C for 30 min. The absorbance was measured at 560 nm with the above-mentioned microplate reader. All measurements were performed in triplicate on microplates.

The creatinine concentration of the urine was determined by the Jaffe method using LabAssay™ Creatinine (Wako Pure Chemical Industries Ltd., Osaka, Japan). All measurements were performed in triplicate on microplates. Values for urinary HEL, 8-OHdG, DT, and H_2_O_2_ were normalized by each value of creatinine measured in the urine. 

### 2.4. Data Analysis 

The differences in levels of urinary oxidative stress biomarkers between individuals in groups of low V/F intake and higher V/F intake were assessed using a Mann*–*Whitney U test. We also carried out a multiple regression analysis to evaluate the independent relationship between V/F intake and oxidative stress biomarkers, with adjustment for potential confounders. Statistical analysis was carried out using IBM-SPSS for Windows ver. 26.0 (SPSS Inc., Chicago, IL, USA). 

## 3. Results 

Table 1 summarizes the characteristics of the study population for age, BMI, education, awareness of the current recommendations for daily V/F intake, self-reported daily V/F intake, habit of physical exercise, and biomarker levels in the urine. We found only 5.8% of participants were aware of the current daily intake recommendations for vegetables, and 1.9% for fruit. In addition, only 19.4% and 23.3% reported that they consumed the recommended amounts of 350 g/day or more of vegetables and 200 g/day or more of fruit, respectively, according to their self-reported daily V/F intake. More than two-thirds of the participants consumed less than 250 g of vegetables and almost one-third of the participants consumed less than 100 g of fruit each day. About 70% of the participants engaged in physical exercise at least once a week. 

As shown in Table 2, individuals who consumed less fruit (<100 g/day) or vegetables (<250 g/day) had significantly higher HEL excretion in their urine than those who consumed higher levels of fruit (≥100 g/day) or vegetables (≥250 g/day); urinary 8-OHdG excretion was also higher in participants who consumed less fruit and vegetables, though the differences were not statistically significant. 

Urinary DT excretion did not differ between the lower and higher fruit and vegetable consumption groups (Table 2). To our surprise, H_2_O_2_ levels in the urine were significantly higher in the groups with higher fruit or vegetable consumption.

The findings, based on the results of the multiple regression analysis, showed that vegetable consumption was an important inhibiting factor of early lipid peroxidation, measured as HEL in urine, independent of age, BMI, frequency of physical exercise per week, and amount of fruit consumption (Table 3), i.e., the more vegetables the participants consumed, the lower the levels of HEL excreted in their urine. This model explained 6% of the variation of HEL in the urine. According to the results of the multiple regression analysis (Table 3), we did not observe any influential effects of V/F consumption on H_2_O_2_ excretion in urine after adjustment for age, BMI, frequency of physical exercise per week, and amount of fruit or vegetable consumption. 

However, vegetable consumption was an influential factor for urinary DT after adjustment for age, BMI, frequency of physical exercise per week, and the amount of fruit consumption, based on the results of a multiple regression analysis. The model explained 4.7% of the variation of DT in the urine (Table 3). We also found the oxidative biomarker levels of HEL, 8-OHdG, and DT in the urine were significantly lower among the participants who engaged in physical exercise at least once a week (Table 4). 

The findings did not show any significant correlation between BMI with the urinary biomarkers and V/F consumption (Table 5).

## 4. Discussion

The evidence that individuals who consumed less fruit or vegetables showed higher excretion of HEL and 8-OHdG in their urine is supported by several studies performed in adults, in which higher amounts of vegetable or fruit consumption showed reduced levels of urinary HEL and 8-OHdG [31,32,33], implying an increase in fruit/vegetable consumption may play an important role in diminishing oxidative cellular damage to lipids and DNA in the body. 

Atherosclerosis has been proven to begin in childhood and youth [40,41,42]. Studies by the Pathobiological Determinants of Atherosclerosis in Youth (PDAY) Research Group indicated that serum lipoprotein cholesterol levels are important determinants of the early stages of atherosclerosis in adolescents [42]. HEL, produced by the reaction of linoleic acid hydroperoxide and lysine residual, is considered as a useful biomaker of capturing early products of lipid peroxidation and protein modification in humans [7,43]. HEL production was raised by oxidatively modified low-density lipoproteins in atherosclerotic rabbits [10].

Vegetables and fruit, especially vegetables, have been reported to have antioxidant properties through various possible mechnisms, such as “free-radical scavenging activity”, “transition-metal-chelating activity”, and singlet-oxygen-quenching capacity [44,45,46]. Some studies have found sulforaphane (SFN), mainly contained in cruciferous vegetables (such as broccoli, Brussels sprouts, cabbage, cauliflower, kale), decreased the oxidative biomarker levels of lipid peroxidation (MDA) and DNA modification (8-OHdG) in the ROS-induced neonatal hypoxia-ischemia brain of rats, and as well decreased the generation of ROS and MDA and upregulated the antioxidative related enzyme expressions (Glutathione S-transferase, Glutathione peroxidase, and thioredoxin reductase) in human vascular endothelial EA.hy.926 cells, through the activation of nuclear factor-erythroid 2-related factor-2 (Nrf2) [47,48].

A systematic review of 95 prospective studies by a recent meta-analysis [49] demonstrated that an increase in the consumption of fruit, vegetables, and fruit and vegetables combined, of 200 g per day, significantly decreased the relative risk of coronary heart disease, stroke, total cancers, and all-cause mortality. Xie et al. found increased serum levels of superoxide dismutase (SOD) and glutathione peroxidase (GPx) and decreased levels of MDA in serum and 8-OHdG in the urine of coke oven workers with the highest quartile dietary intake of fruit and vegetables in comparison with those in the lowest quartile [50]. However, some contrary results are also reported in the literature. An intervention for low fruit/vegetable consumers, using a diet supplemented with additional fruits and vegetables for 12 weeks [34], did not show significant effects on the lymphocyte DNA damage of the participants. Briviba et al. [51] also found no differences in DNA damage after a 12-week vegetable and fruit intervention in male nonsmokers. These results might imply that the effects of V/F consumption against ROS-induced oxidative stress do not necessarily appear after short-term intervention trials (12 weeks). In other words, habitual consumption of V/F possibly plays an important role in the attenuation of ROS-induced oxidative modification in the body. 

The current study found higher urinary H_2_O_2_ excretion in the group with higher fruit or vegetable consumption. Akagawa et al. found that polyphenols had H_2_O_2_-generating properties under quasi-physiological conditions [52]; it has been reported that some fruits and vegetables have shown high polyphenol contents, such as radish, burdock, celery, blueberry, sweet cherry, and apple [53]. However in the literature, there is no evidence that the consumption of polyphenol-rich V/F could result in higher level of H_2_O_2_ ecretion in urine, although several researchers reported coffee drinking artefactually increased the urinary excretion of H_2_O_2_ in a group of healthy individuals because of the hydroxyhydroquinone (HHQ) contained in coffee beans; autoxidation of HHQ in the urine can generate H_2_O_2_ dose-dependently [54,55], whereas the findings on green tea and green tea extract had contradictory results for urinary H_2_O_2_ excretion [55,56]. In addition, a multiple regression analysis did not observe any influential effects of V/F consumption on the H_2_O_2_ excretion in urine after adjustment for age, BMI, frequency of physical exercise per week, and the amount of fruit or vegetable consumption. Further investigation is needed to confirm the association between V/F consumption and the level of H_2_O_2_ excretion in urine.

It is known that DT is one of the biomarkers of the oxidation of protein [12]. Studies in vitro have demonstrated that radical-like intermediates can abstract atomic hydrogen (H) from tyrosine residuals in proteins and then covalently cross-link tyrosine residuals into DT [57]. In addition, DT release has been suggested not only as a biomarker for protein oxidation but also as “an endogenous marker for the selective degradation of oxidatively modified proteins” [58]. So far there are no reports regarding whether vegetable consumption contributes to the cross-linking of tyrosine residuals or to the degradation of oxidatively modified protein in the body. Kato et al. [59] found polyphenols, such as ferulic acid, gallic acid, and quercetin, inhibited DT formation in vitro, whereas a report by Izabela et al. did not find a protective effect of polyphenols against DT formation in vitro [60]. Twelve weeks of intake of cocoa powder, rich in catechins and procyanidins, showed a decrease in the urinary DT of a small sample of Japanese male subjects [61]. Several reports have demonstrated that DT content was significantly higher in the aortic tissue of hyperglycemic animals [62], and that urinary DT excretion was increased in people with diabetes [63], and in children with an autism spectrum disorder [64]. It is considered that protein contained in food can be oxidized when cooked, and low levels of DT, as well nitrotyrosine and o-tyrosine are found in many normal body tissues [19,65]. Why urinary DT level was associated with higher vegetable consumption in the current study is unclear. Further studies with larger sample sizes are needed to confirm the current findings. We did not find any association between levels of fruit consumption and H_2_O_2_ excretion in urine. 

The findings of lower urinary HEL, 8-OHdG, and DT among the participants who engaged in physical exercise at least once a week are partially supported by an animal study which demonstrated lower levels of DT in urine and higher levels of SOD and GPx of exercising rats in comparison with the sedentary group [66]. It has been reported that elevated urinary MDA, 8-OHdG, and DT levels were measured on the same day as one hour of exercise was undertaken (cycle ergometer) [67]; however, several works have indicated regular moderate physical exercise can increase the activities of some antioxidant enzymes, such as superoxide dismutase, glutathione peroxidase, and catalase in human muscle [68,69,70]. Therefore, the formation of healthy lifestyles, such as habitual V/F intake and physical exercise in the early lifecycle may be expected to modulate the oxidative–antioxidative balance in the body.

Several limitations should be taken into account when interpreting the current findings as follows: (1) The study was a cross-sectional design limited to a relatively small number of teenaged girls; therefore, caution should be taken to avoid generalizing the findings. Future study with a relatively large sample size is needed to better elucidate the relation of V/F consumption to the generation of oxidative stress biomarkers in urine. (2) Data on daily energy intake and tea consumption of the participants was not available; this might affect the magnitude of the relationship between V/F consumption and urinary levels of oxidative biomarkers. (3) Reporting bias might be introduced by the self-reported measure of daily V/F consumption. (4) We did not include male teenagers in the current study; future studies should include both genders to examine the gender differences in the association of V/F consumption with oxidative biomarker levels. Despite these limitations, the current findings indicate the important role of V/F intake in the alteration of oxidative biomarker levels in urine.

## 5. Conclusions

The decrease in HEL production reflecting early lipid peroxidation and protein modification in groups with regularly higher vegetable intake found in the current results may suggest that relatively higher consumption of vegetables would help the prevention of early lipid peroxidation and protein modification in adolescents. The findings also imply the significance that promoting healthy lifestyles should start at early stage of life for the prevention of atherosclerosis. Further studies with large subjects are needed to confirm the effects of vegetable consumption on the inhibition of early lipid peroxidation and protein modification.

## Figures and Tables

**Table 1:** Participant characteristics.

			mean ± SD (n)
Age			15.6 ± 1.7 (103)
BMI			20.7 ± 2.3 (103)
			***n* (%)**
Education			
Junior high school students			33 (32.0)
High school students			70 (68.0)
Awareness of the recommended amount of daily vegetable intake			
Yes			6 (5.8)
No			97 (94.2)
Awareness of the recommended amount of daily fruit intake			
Yes			2 (1.9)
No			101 (98.1)
Self–reported daily vegetable intake (g/day)			
<250			72 (69.9)
250–349			11 (10.7)
≥350			20 (19.4)
Self–reported daily fruit intake (g/day)			
<100			32 (31.1)
100–199			47 (45.6)
≥200			24 (23.3)
Physical exercise at least once a week			
Yes			72 (69.9)
No			31 (30.1)
Oxidative biomarkers in urine			median (min, max)
HEL (nmol/g Cr)			86.22 (10.38, 884.71)
DT (µmol/g Cr)			2.55 (0.56, 11.2)
8-OHdG (ng/mg Cr)			7.29 (2.22, 23.21)
H2O2 (µmol/g Cr)			45.85 (0.5, 281.06)

HEL: hexanoyl-lysine. DT: dityrosine.

**Table 2:** Urinary biomarker concentrations by daily consumption of fruit and vegetables.

	**Fruit**		
	<100 g/d (*n* = 32)	≥100 g/d (*n* = 71)	***p* Value**
HEL (nmol/g Cr)	95.09 (835.22)	79.24 (379.04)	0.033
8-OHdG (ng/mg Cr)	7.90 (15.46)	7.07 (20.81)	0.127
DT (μmol/g Cr)	2.81 (6.18)	2.41 (10.42)	0.385
H_2_O_2_ (µmol/g Cr)	40.14 (249.33)	57.88 (277.55)	0.092
	**Vegetable**		
	<250 g/d (*n* = 72)	≥250 g/d (*n* = 31)	***p* Value**
HEL (nmol/g Cr)	89.86 (874.33)	70.33 (222.69)	0.052
8-OHdG (ng/mg Cr)	7.41 (20.99)	7.01 (15.29)	0.278
DT (μmol/g Cr)	2.45 (10.63)	2.80 (5.82)	0.774
H_2_O_2_ (µmol/g Cr)	43.27 (280.56)	69.31 (198.99)	0.349

Data are median (range) and analyzed by Mann-Whitney U test.

**Table 3:** Multiple regression analysis of fruit and vegetable intake and urinary biomarkers.

	Vegetable Intake		Fruit Intake		Adjusted R^2^ ^a^
	β	*p*	β	*p*	
HEL	−0.332	0.014	0.171	0.213	0.060
8-OHdG	0.002	0.987	0.106	0.437	0.067
DT	0.302	0.026	−0.019	0.890	0.047
H_2_O_2_	0.121	0.253	0.039	0.715	0.089

Urinary biomarker levels and amount of fruit and vegetable intake, log-transformed; ^a^ adjusted for age, BMI, frequency of exercise per week, and amount of vegetable or fruit intake.

**Table 4:** Urinary biomarker concentrations by physical exercise.

	Physical Exercise at Least Once a Week		*p* Value
	No (*n* = 31)	Yes (*n* = 72)	
Urinary HEL (nmol/g Cr)	140.7 (32.3, 352.6)	71.2(10.4, 884.7)	0.002
Urinary 8-OHdG (ng/mg Cr)	10.0 (4.2, 23.2)	6.6 (2.2, 15.4)	<0.001
Urinary DT (µmol/g Cr)	3.6 (0.6, 11.2)	2.2 (0.8, 5.5)	<0.001
Urinary H_2_O_2_ (µmol/g Cr)	42.3 (0.5, 249.8)	47.2 (9.7, 281.1)	0.930

Data are median (min, max) and analyzed by a Mann-Whitney U test.

**Table 5:** The relationship between BMI with the urinary biomarkers and fruit/vegetable intake.

	BMI (kg/m^2^)	
	*r*	*p* Value
Fruit intake (g/day)	−0.015	0.878
Vegetable intake (g/day)	−0.125	0.211
HEL (nmol/g Cr)	−0.094	0.348
8-OHdG (ng/mg Cr)	−0.149	0.136
DT (μmol/g Cr)	−0.056	0.573
H_2_O_2_ (µmol/g Cr)	−0.033	0.745

Data are analyzed by a Spearman correlation analysis.

## Data Availability

Not applicable.

## References

[1] Halliwell B., Gutteridge J.M.C., Halliwell B., Gutteridge J.M.C. (1999). Oxidative stress. Free Radicals in Biology and Medicine.

[2] Sies H. (1991). Oxidative stress: From basic research to clinical application. Am. J. Med..

[3] Committee on Biological Markers of the National Research Council (1987). Biological markers in environmental health research. Environ. Health Perspect..

[4] Bonassi S., Au W.W. (2002). Biomarkers in molecular epidemiology studies for health risk prediction. Mutat. Res..

[5] Ogino K., Wang D.-H. (2007). Biomarkers of Oxidative/Nitrosative Stress: An Approach to Disease Prevention. Acta Med. Okayama.

[6] Giulivi C., Davies K.J.A., Packer L. (1994). Dityrosine: A marker for oxidatively modified proteins and selective proteolysis. Methods in Enzymology, Oxygen Radicals in Biological Systems Part C.

[7] Kato Y., Mori Y., Makino Y., Morimitsu Y., Hiroi S., Ishikawa T., Osawa T. (1999). Formation of Nepsilon-(hexanonyl)lysine in protein exposed to lipid hydroperoxide. A plausible marker for lipid hydroperoxide-derived protein modification. J. Biol. Chem..

[8] Cooke M.S., Evans M.D., Herbert K.E., Lunec J. (2000). Urinary 8-oxo-2′-deoxyguanosine—Source, significance and supplements. Free Radic. Res..

[9] Kato Y., Wu X., Naito M., Nomura H., Kitamoto N., Osawa T. (2000). Immunochemical detection of protein dityrosine in atherosclerotic lesion of apo-E-deficient mice using a novel monoclonal antibody. Biochem. Biophys. Res. Commun..

[10] Naito M., Wu X., Nomura H., Kodama M., Kato Y., Kato Y., Osawa T. (2002). The protective effects of tetrahydrocurcumin on oxidative stress in cholesterol-fed rabbits. J. Atheroscler. Thromb..

[11] Ghezzo A., Visconti P., Abruzzo P.M., Bolotta A., Ferreri C., Gobbi G., Malisardi G., Manfredini S., Marini M., Nanetti L. (2013). Oxidative stress and erythrocyte membrane alterations in children with autism: Correlation with clinical features. PLoS ONE.

[12] Heinecke J.W., Li W., Daehnke H.L., Goldstein J.A. (1993). Dityrosine, a specific marker of oxidation, is synthesized by the myeloperoxidase-hydrogen peroxide system of human neutrophils and macrophages. J. Biol. Chem..

[13] Kanauchi M., Nishioka H., Hashimoto T. (2002). Oxidative DNA damage and tubulointerstitial injury in diabetic nephropathy. Nephron.

[14] Akagi S., Nagake Y., Kasahara J., Sarai A., Kihara T., Morimoto H., Yano A., Nakao K., Nanba K., Ichikawa H. (2003). Significance of 8-hydroxy-2′-deoxyguanosine levels in patients with chronic renal failure. Nephrology.

[15] Chiou C.C., Chang P.Y., Chan E.C., Wu T.L., Tsao K.C., Wu J.T. (2003). Urinary 8-hydroxydeoxyguanosine and its analogs as DNA marker of oxidative stress: Development of an ELISA and measurement in both bladder and prostate cancers. Clin. Chim. Acta.

[16] Loft S., Poulsen H.E., Vistisen K., Knudsen L.E. (1999). Increased urinary excretion of 8-oxo-2′-deoxyguanosine, a biomarker of oxidative DNA damage, in urban bus drivers. Mutat. Res..

[17] Barbato D.L., Tomei G., Tomei F., Sancini A. (2010). Traffic air pollution and oxidatively generated DNA damage: Can urinary 8-oxo-7,8-dihydro-2-deoxiguanosine be considered a good biomarker? A meta-analysis. Biomarkers.

[18] Lee M.W., Chen M.L., Lung S.C., Tsai C.J., Yin X.J., Mao I.F. (2010). Exposure assessment of PM2.5 and urinary 8-OHdG for diesel exhaust emission inspector. Sci. Total Environ..

[19] Halliwell B., Gutteride J.M.C., Halliwell B., Gutteride J.M.C. (2015). Oxidative stress and redox regulation: Adaptation, damage, repair, senescence, and death. Free Radicals in Biology and Medicine.

[20] Schreck R., Rieber P., Baeuerle P.A. (1991). Reactive oxygen intermediates as apparently widely used messengers in the activation of the NF-kappa B transcription factor and HIV-1. EMBO J..

[21] Veal E.A., Day A.M., Morgan B.A. (2007). Hydrogen Peroxide Sensing and Signaling. Mol. Cell.

[22] Mathru M., Rooney M.W., Dries D.J., Hirsch L.J., Barnes L., Tobin M.J. (1994). Urine hydrogen peroxide during adult respiratory distress syndrome in patients with and without sepsis. Chest.

[23] Campos C., Guzmán R., López-Fernández E., Casado A. (2011). Evaluation of urinary biomarkers of oxidative/nitrosative stress in adolescents and adults with Down syndrome. Biochim. Biophys. Acta..

[24] Banerjee D., Jacob J., Kunjamma G., Madhusoodanan U.K., Ghosh S. (2004). Measurement of urinary hydrogen peroxide by FOX-1 method in conjunction with catalase in diabetes mellitus—A sensitive and specific approach. Clin. Chim. Acta.

[25] Wallace T.C., Bailey R.L., Blumberg J.B., Burton-Freeman B., Chen C.O., Crowe-White K.M., Drewnowski A., Hooshmand S., Johnson E., Lewis R. (2020). Fruits, vegetables, and health: A comprehensive narrative, umbrella review of the science and recommendations for enhanced public policy to improve intake. Crit. Rev. Food Sci. Nutr..

[26] Lampe J.W. (1999). Health effects of vegetables and fruit: Assessing mechanisms of action in human experimental studies. Am. J. Clin. Nutr..

[27] Boeing H., Bechthold A., Bub A., Ellinger S., Haller D., Kroke A., Leschik-Bonnet E., Müller M.J., Oberritter H., Schulze M. (2012). Critical review: Vegetables and fruit in the prevention of chronic diseases. Eur. J. Nutr..

[28] National Institute of Health and Nutrition Outline for the Results of the National Health and Nutrition Survey Japan. https://www.mhlw.go.jp/content/10904750/000351576.pdf.

[29] (2000). Ministry of Health, Labour and Welfare, Japan. Health Japan 21 (The National Health Promotion Movement in 21st Century). https://www.mhlw.go.jp/www1/topics/kenko21_11/pdf/all.pdf.

[30] (2005). Ministry of Agriculture, Forestry and Fisheries, Japan. Guidelines for Balanced Diet. https://www.mhlw.go.jp/bunya/kenkou/pdf/eiyou-syokuji8.pdf.

[31] Verhagen H., de Vries A., Nijhoff W.A., Schouten A., van Poppel G., Peters W.H., van den Berg H. (1997). Effect of Brussels sprouts on oxidative DNA-damage in man. Cancer Lett..

[32] Young J.F., Nielsen S.E., Haraldsdóttir J., Daneshvar B., Lauridsen S.T., Knuthsen P., Crozier A., Sandström B., Dragsted L.O. (1999). Effect of fruit juice intake on urinary quercetin excretion and biomarkers of antioxidative status. Am. J. Clin. Nutr..

[33] Iwasawa H., Morita E., Yui S., Yamasaki M. (2011). Anti-oxidant Effects of Kiwi Fruit in Vitro and in Vivo. Biol. Pharm. Bull..

[34] Duthie S.J., Duthie G.G., Russell W.R., Kyle J.A.M., Macdiarmid J.I., Rungapamestry V., Stephen S., Megias-Baeza C., Kaniewska J.J., Shaw L. (2018). Effect of increasing fruit and vegetable intake by dietary intervention on nutritional biomarkers and attitudes to dietary change: A randomised trial. Eur. J. Nutr..

[35] Francina R., Baldrick J., Elborn S., Woodside J.V., Treacy K., Bradley J.M., Patterson C.C., Schock B.C., Ennis M., Young I.S. (2012). Effect of fruit and vegetable intake on oxidative stress and inflammation in COPD: A randomised controlled trial. Eur. Respir. J..

[36] Havas S., Treiman K., Langenberg P., Ballesteros M., Anliker J., Damron D., Feldman R. (1998). Factors associated with fruit and vegetable consumption among women participating in WIC. J. Am. Diet. Assoc..

[37] Wang D.-H., Kogashiwa M., Mori N., Yamashita S., Fujii W., Ueda N., Homma H., Suzuki H., Masuoka N. (2016). Psychosocial Determinants of Fruit and Vegetable Consumption in a Japanese Population. Int. J. Environ. Res. Public Health.

[38] Saito S., Yamauchi H., Hasui Y., Kurashige J., Ochi H., Yoshida K. (2000). Quantitative determination of urinary 8-hydroxydeoxyguanosine (8-OH-dg) by using ELISA. Res. Commun. Mol. Pathol. Pharmacol..

[39] Sato Y., Ogino K., Sakano N., Wang D.-H., Yoshida J., Akazawa Y., Kanbara S., Inoue K., Kubo M., Takahashi H. (2013). Evaluation of urinary hydrogen peroxide as an oxidative stress biomarker in a healthy Japanese population. Free Radic. Res..

[40] Strong J.P., McGill H.C. (1963). The nature history of aortic atherosclerosis: Relationship to race, sex, and coronary lesions in New Orleans. Exp. Mol. Pathol..

[41] McGill H.C. (1968). Fatty streaks in the coronary arteries and aorta. Lab. Investig..

[42] Pathobiological Determinants of Atherosclerosis in Youth (PDAY) Research Group (1990). Relationship of atherosclerosis in young men to serum lipoprotein cholesterol concentrations and smoking. A preliminary report from the Pathobiological Determinants of Atherosclerosis in Youth (PDAY) Research Group. JAMA.

[43] Sakai K., Kino S., Masuda A., Takeuchi M., Ochi T., Osredkar J., Rejc B., Gersak K., Ramarathnam N., Kato Y., Kato Y. (2014). Determination of HEL (Hexanoyl-Lysine Adduct): A novel biomarker for omega-6 PUFA oxidation. Lipid Hydroperoxide-Derived Modification of Biomolecules.

[44] Číž M., Čížová H., Denev P., Kratchanova M., Slavov A., Lojek A. (2010). Different methods for control and comparison of the antioxidant properties of vegetables. Food Control.

[45] Lotito S.B., Frei B. (2006). Consumption of flavonoid-rich foods and increased plasma antioxidant capacity in humans: Cause, consequence, or epiphenomenon?. Free Radic. Biol. Med..

[46] Shan B., Cai Y.Z., Sun M., Corke H. (2005). Antioxidant capacity of 26 spice extracts and characterization of their phenolic constituents. J. Agric. Food Chem..

[47] Ping Z., Liu W., Kang Z., Cai J., Wang Q., Cheng N., Wang S., Wang S., Zhang J.H., Sun X. (2010). Sulforaphane protects brains against hypoxic-ischemic injury through induction of Nrf2-dependent phase 2 enzyme. Brain Res..

[48] Li B., Tian S., Liu X., He C., Ding Z., Shan Y. (2015). Sulforaphane protected the injury of human vascular endothelial cell induced by LPC through up-regulating endogenous antioxidants and phase II enzymes. Food Funct..

[49] Aune D., Giovannucci E., Boffetta P., Fadnes L.T., Keum N., Norat T., Greenwood D.C., Riboli E., Vatten L.J., Tonstad S. (2017). Fruit and vegetable intake and the risk of cardiovascular disease, total cancer and all-cause mortality—A systematic review and dose-response meta-analysis of prospective studies. Int. J. Epidemiol..

[50] Xie Z., Lin H., Fang R., Shen W., Li S., Chen B. (2015). Effects of a fruit-vegetable dietary pattern on oxidative stress and genetic damage in coke oven workers: A cross-sectional study. Environ. Health.

[51] Briviba K., Bub A., Möseneder J., Schwerdtle T., Hartwig A., Kulling S., Watzl B. (2008). No differences in DNA damage and antioxidant capacity between intervention groups of healthy, nonsmoking men receiving 2, 5, or 8 servings/day of vegetables and fruit. Nutr. Cancer.

[52] Akagawa M., Shigemitsu T., Suyama K. (2003). Production of hydrogen peroxide by polyphenols and polyphenol-rich beverages under quasi-physiological conditions. Biosci. Biotechnol. Biochem..

[53] Sakakibara H., Honda Y., Nakagawa S., Ashida H., Kanazawa K. (2003). Simultaneous determination of all polyphenols in vegetables, fruits, and teas. J. Agric. Food Chem..

[54] Long L.H., Halliwell B. (2000). Coffee drinking increases levels of urinary hydrogen peroxide detected in healthy human volunteers. Free Radic. Res..

[55] Hiramoto K., Kida T., Kikugawa K. (2002). Increased urinary hydrogen peroxide levels caused by coffee drinking. Biol. Pharm. Bull..

[56] Halliwell B., Long L.H., Yee T.P., Lim S., Kelly R. (2004). Establishing biomarkers of oxidative stress: The measurement of hydrogen peroxide in human urine. Curr. Med. Chem..

[57] Giulivi C., Traaseth N.J., Davies K.J.A. (2003). Tyrosine oxidation products: Analysis and biological relevance. Amino Acids.

[58] Giulivi C., Davies K.J. (1993). Dityrosine and tyrosine oxidation products are endogenous markers for the selective proteolysis of oxidatively modified red blood cell hemoglobin by (the 19 S) proteasome. J. Biol. Chem..

[59] Kato Y., Nagao A., Terao J., Osawa T. (2003). Inhibition of myeloperoxidase-catalyzed tyrosylation by phenolic antioxidants in vitro. Biosci. Biotechnol. Biochem..

[60] Izabela S.-B., Sabina G., Grzegorz B. (2014). P78—Polyphenols protect against protein glycoxidation. Free Radic. Biol. Med..

[61] Baba S., Osakabe N., Kato Y., Natsume M., Yasuda A., Kido T., Fukuda K., Muto Y., Kondo K. (2007). Continuous intake of polyphenolic compounds containing cocoa powder reduces LDL oxidative susceptibility and has beneficial effects on plasma HDL-cholesterol concentrations in humans. Am. J. Clin. Nutr..

[62] Pennathur S., Wagner J.D., Leeuwenburgh C., Litwak K.N., Heinecke J.W. (2001). A hydroxyl radical-like species oxidizes cynomolgus monkey artery wall proteins in early diabetic vascular disease. J. Clin. Investig..

[63] Kato Y., Dozaki N., Nakamura T., . Kitamoto N., Yoshida A., Naito M., Kitamura M., Osawa T. (2009). Quantification of modified tyrosines in healthy and diabetic human urine using liquid chromatography/tandem mass spectrometry. J. Clin. Biochem. Nutr..

[64] Anwar A., Abruzzo P.M., Pasha S., Rajpoot K., Bolotta A., Ghezzo A., Marini M., Posar A., Visconti P., Thornalley P.J. (2018). Advanced glycation endproducts, dityrosine and arginine transporter dysfunction in autism—Aa source of biomarkers for clinical diagnosis. Mol. Autism..

[65] Halliwell B., Gutteride J.M.C., Halliwell B., Gutteride J.M.C. (2015). Measurement of reactive species. Free Radicals in Biology and Medicine.

[66] Leeuwenburgh C., Hansen P.A., Holloszy J.O., Heinecke J.W. (1999). Oxidized amino acids in the urine of aging rats: Potential markers for assessing oxidative stress in vivo. Am. J. Physiol..

[67] Orhan H., van Holland B., Krab B., Moeken J., Vermeulen N.P., Hollander P., Meerman J.H. (2004). Evaluation of a multi-parameter biomarker set for oxidative damage in man: Increased urinary excretion of lipid, protein and DNA oxidation products after one hour of exercise. Free Radic. Res..

[68] Leeuwenburgh C., Heinecke J.W. (2001). Oxidative stress and antioxidants in exercise. Curr. Med. Chem..

[69] Ristow M., Zarse K., Oberbach A., Klöting N., Birringer M., Kiehntopf M., Stumvoll M., Kahn C.R., Blüher M. (2009). Antioxidants prevent health-promoting effects of physical exercise in humans. Proc. Natl. Acad. Sci. USA.

[70] He F., Li J., Liu Z., Chuang C.-C., Yang W., Zuo L. (2016). Redox mechanism of reactive oxygen species in exercise. Front. Physiol..

